# The Role of Stressful Parenting and Mineralocorticoid Receptor Haplotypes on Social Development During Adolescence and Young Adulthood

**DOI:** 10.1007/s10964-019-00988-2

**Published:** 2019-02-25

**Authors:** H. M. Endedijk, S. A. Nelemans, R. R. Schür, M. P. Boks, P. van Lier, W. Meeus, C. H. Vinkers, R. A. Sarabdjitsingh, S. Branje

**Affiliations:** 1 Youth and Family, Utrecht University, Utrecht, The Netherlands; 2 Brain Center Rudolf Magnus, University Medical Center Utrecht (UMCU), Utrecht, The Netherlands; 3 Clinical Developmental Psychology, Vrije Universiteit, Amsterdam, The Netherlands; 4 Developmental Psychology, Tilburg University, Tilburg, The Netherlands; 5 Department of Psychiatry, Amsterdam UMC (location VUmc), Amsterdam, The Netherlands; 6 Department of Anatomy and Neurosciences, Amsterdam UMC (location VUmc), Amsterdam, The Netherlands

**Keywords:** Mineralocorticoid Receptor, Prosocial behavior, Empathy, Stress, Adolescence, Parenting

## Abstract

The development of social behavior could be affected by stressful parenting. The mineralocorticoid receptor, one of the two main receptors for the stress hormone cortisol, plays a vital role in adequate responses to stress. Therefore, the effects of stressful parenting on social development (i.e., empathic concern, perspective taking and prosocial behavior) may be moderated by functional genetic variation in mineralocorticoid receptor haplotypes (a combination of alleles). A group of 343 adolescents (44.3% females) was followed from the age of 13 until 24 years. Growth curve analyses showed lower levels of prosocial behaviors and a slower increase in empathic concern and perspective taking in adolescents who reported more stressful parenting. In contrast, relatively higher levels of prosocial behavior, empathic concern and perspective taking were present in combination with stress resilient mineralocorticoid receptor haplotypes. Despite sex differences in social development with earlier social development for girls, no consistent sex differences were found with regard to mineralocorticoid receptor haplotypes. The current study showed that genetic variation in mineralocorticoid receptor impacts the social development during adolescence and young adulthood.

## Introduction

Adolescents increasingly become better in perspective taking (Tousignant et al. 2017), empathic concern (van der Graaff et al. 2014) and show more prosocial behavior (van der Graaff et al. 2018). Environmental stress, like abusive parenting, is supposed to influence this social development (Sandi and Haller 2015). Higher levels of environmental stress are related to less empathy and increased aggression and antisocial behavior via psychobiological processes (Susman 2006). One of the biological factors that could play a role in the relation between environmental stress and the development of social behavior is the mineralocorticoid receptor (MR) (De Kloetet al. 2005) as it is closely involved in the appraisal of a stressful situation and it is one of the two receptors for the stress hormone cortisol (ter Heegde et al. 2015). Experimental studies have shown that pharmacological stimulation of MR resulted in enhanced empathic concern in clinically depressed adult patients (Wingenfeld et al. 2014) and enhanced emotion processing in a healthy sample (Schultebraucks et al. 2016). Therefore, the aim of this study was to investigate whether genetic variation in MR would moderate the effects of stressful parenting on the social development (i.e., prosocial behavior, empathic concern and perspective taking development) from adolescence to young adulthood in a community sample.

During adolescence, social behavior changes (Forbes and Dahl 2010) due to development in social information processing (Nelson et al. 2005). While certain social cognitive processes, like prosocial reasoning (Eisenberg et al. 2015) and social knowledge (Tousignant et al. 2017), are already well developed during childhood, more complex aspects of social cognition increase over the course of adolescence. Mainly adolescents’ perspective taking undergoes quick development (van der Graaff et al. 2014), which enables adolescents to attribute mental states such as beliefs, desires and intentions to others (Blakemore and Choudhury 2006). Similarly, empathic concern and emotion processing show strong development during adolescence (Tousignant et al. 2017). A meta-analysis on prosocial behavior has shown an increase only in early adolescence until the age of 16 and no development in late adolescence (Fabes et al. 1999). This is confirmed in a recent longitudinal adolescent study that even found a decreasing trend in prosocial behavior in late adolescence (van der Graaff et al. 2018), although other studies showed decreasing trends over the whole course of adolescence (e.g., Luengo Kanacri et al. 2013). Interestingly, social development seems to differ between boys and girls. van der Graaff et al. (2014) found that girls already reached adult-levels of empathic concern in the beginning of adolescence, while boys developed empathic concern over the whole course of adolescence. Also, perspective taking and prosocial behavior seems to develop differently in boys and girls, mainly in early and mid-adolescence, with growth starting earlier for girls than for boys (van der Graaff et al. 2018).

Adolescence is a period in which the long-lasting effects of earlier exposure to physical or emotional abuse and neglect become evident (Lupien et al. 2009). Stress models suggest that early life stress, like stressful parenting, results in decreased levels of social motivation, reduced social behaviors, increased aggressiveness, and stronger development of antisocial characteristics (Sandi and Haller 2015). A form of stressful parenting that could be related to social development in adolescence is childhood trauma like sexual, physical, or emotional abuse or neglect. Indeed, stress in humans has been related to less empathy and increased aggression and antisocial behavior (Susman 2006).

Also, psychological control is a form of abusive parenting (Del Giudice et al. 2011) that could hinder the social development of adolescents. Psychological control involves attempts that intrude or manipulate the thinking processes, self-expression, and emotions of the child (Barber 1996). According to the self-determination theory (Deci and Ryan 2000), such a controlling environment with conditional love of parents lead to negative expectations of interpersonal relationships, which elicit maladaptive social behaviors and impairments in social development (Soenens and Vansteenkiste 2010). Mainly during adolescence, psychological control can be intrusive, as adolescent have an increasing need for autonomy (Lansford et al. 2014). Therefore, parental psychological control during adolescence can be considered as stressful parenting, which could affect the social development of adolescents. Indeed, parental psychological control has been related to lower levels of social behavior and higher levels of relational aggression in adolescents (Loukas et al. 2005) and emerging adults (Clark et al. 2015).

Besides differences in stressful parenting, adolescents also differ in their general stress reactivity (Ellis et al. 2013). Resiliency to the negative consequences of stress (McEwen et al. 2015) may be explained by genetic variation. Genetic variations important in this regard are the MR-2G/C (rs2070951) and MRI180V (rs5522), which are single nucleotide polymorphisms (SNPs) that both affect in vitro transactivation by altering Mineralocorticoid Receptor (MR) expression or functionality (ter Heegde et al. 2015). Thereby, these SNPs affect the stress response (van Leeuwen et al. 2011). Upon experiencing stress, the Hypothalamic-Pituitary-Adrenal axis (HPA-axis) becomes active and releases several hormones to deal with the stressor, including cortisol (Del Giudice et al. 2011). Cortisol binds to MR in the brain, thereby providing negative feedback on the HPA-axis which subsequently returns to the prestress state after the stressor is gone (Joëls and de Kloet 2017).

Previously, it has been found that MR haplotypes (based on two SNPs rs5522 and rs2070951) differentially affect MR activity and expression and thereby the functionality, as they affect the maximal transactivation and protein expression (ter Heegde et al. 2015). The haplotype coined “CA” is associated with increased MR expression and activity, leading to a more reactive HPA-axis with lower basal non-stress levels, and could therefore be advantageous for adolescents’ social behavior (van Leeuwen et al. 2011). Although earlier studies found no relation between MR functioning and perspective taking (Wingenfeld et al. 2014), or even diminished perspective taking (Wingenfeld et al. 2016), higher MR functioning after pharmacological stimulation resulted in enhanced empathic concern (Wingenfeld et al. 2014) in clinically depressed adults. Moreover, in healthy subjects increased emotion processing has been reported after pharmacological stimulation (Schultebraucks et al. 2016), and diminished emotion processing after pharmacological inhibition of MR functioning (Young et al. 2016).

This expected positive effect of the MR CA haplotype could even be stronger for adolescents who experienced stressful parenting. Vinkers et al. (2015) found stronger protective effects of the MR CA haplotype on depression symptoms for adults with higher levels of childhood trauma in a population based sample. As MRs are involved in every stress response, the MR CA haplotype can result in effective termination of each stressor (ter Heegde et al. 2015). In adolescents who experience high levels of environmental stress, the MR CA haplotype can play a large role in the stress regulation of each of these stressful experiences, and thereby in the possible negative consequences of environmental stress. In contrast, in adolescents who experience only incidental daily stressors, the consequences of effective stress regulation might be smaller.

In addition, a growing body of evidence from experimental studies has shown that the effects of MR haplotypes are sex-specific, with the CA haplotype being protective in females but not in males (ter Heegde et al. 2015). The sex-specific MR haplotype effects might be explained by the female hormones progesterone and estrogen that positively affect MR functioning (Carey et al. 1995). These findings suggest that mainly female adolescents with a MR CA haplotype might be resilient to the negative consequences of stress for their social development.

## Current Study

Indicators of social behavior were operationalized by the concepts prosocial behavior, perspective taking and empathic concern. The development of prosocial behavior, empathic concern and perspective taking was examined, in order to study the interaction between stressful parenting and MR on the social development. The aim was to extend the evidence on the role of the MR in indicators of social behavior based on pharmacological modulation by investigating the effects of common functional genetic variation in the MR gene on social development. Stress models suggest that early life stress, like stressful parenting, results in decreased levels of social behaviors (Sandi and Haller 2015). Therefore, the expectation was that stressful parenting, as indicated by high levels of parental psychological control or childhood trauma, would be negatively associated with prosocial behavior, empathic concern and perspective taking during adolescence and young adulthood. Moreover, given that the MR CA haplotype supports stress regulation (van Leeuwen et al. 2011), the hypothesis was that adolescents with more MR CA haplotypes would show higher levels of prosocial behavior, empathic concern and perspective taking during adolescence and young adulthood as compared to individuals with fewer CA haplotypes, particularly in adolescents that experienced stressful parenting. Given the female-specific MR CA haplotype effects in experimental studies (ter Heegde et al. 2015), the positive effect of MR CA haplotype was expected to be stronger in females than males.

## Methods

### Participants

Participants were 343 adolescents (55.7% boys) from the center and west of The Netherlands with a mean age of 13 years (SD = .4) at Wave 1. They all attended the first grade of secondary school at the first wave of data collection in 2006. The adolescents were followed longitudinally until age 24 as part of the longitudinal community study RADAR-Young (Research on Adolescent Development And Relationships). Of the 522 adolescents participating in this study, 417 (80%) volunteered to take part in an intensive lab study during which saliva was collected for genotyping. An additional 74 adolescents were removed due to genotyping exclusion criteria, most of them (54 adolescents) as they were from another ethnic background, according to their genes. Therefore, 343 adolescents were successfully genotyped around age 17 of whom 7.1% were from low SES. All participants were Dutch, except from two coming originally from England. Adolescents from the genetic sample did not differ from adolescents from the total sample on sex, age, parental psychological control, childhood trauma, prosocial behavior, empathic concern, or perspective taking (all *p*s < .05). Attrition rates were low, with 72.3% of the adolescents participating at all waves. Little’s (1988) Missing Completely at Random test showed a nonsignificant χ^2^ (χ^2^/df) of 1.04, indicating no systematic differences between participants with complete data and participants with partially missing data. For the growth curve analysis M*plus* version 8 with Full Information Maximum Likelihood was used to handle missing data (Muthén and Muthén 2017).

### Procedure

Adolescents were visited annually until the age of 18 (6 waves), and biannually for the final 3 waves. During the home visits, adolescents completed questionnaires. For the 9^th^ assessment, the participants completed the questionnaires digitally. Around the 5^th^ wave, DNA was collected by taking saliva samples during a lab assessment. Participants gave active informed consent and for each wave received a small fee.

### Measures

#### Prosocial behavior

Prosocial behavior was measured with the Prosocial Behavior scale, a 10-item subscale of the Dutch version of the Self-report of Aggression and Social Behavior Measure (Morales and Crick 1998). The items have a 7-point Likert scale ranging from 1 (totally not true) to 7 (totally true). An example item is “I try to involve others in conversations”. The internal consistency was high ranging from .83 to .92 across waves.

#### Empathic concern

Empathic concern was measured by the Empathic Concern subscale of the Dutch adjusted version (Hawk et al. 2013) of the Interpersonal Reactivity Index (IRI: Davis 1983). It consists of 7 items on a 5 point Likert-scale ranging from 0 (does not describe me well) to 4 (describes me very well). An example item is “When I see someone being taken advantage of, I feel kind of protective toward them”. The internal consistency was good, ranging from .71 to .78 on the different waves, except for the first wave, on which Cronbach’s alpha was .61.

#### Perspective taking

Perspective taking was measured by the Perspective Taking subscale of the Dutch adjusted version (Hawk et al. 2013) of the Interpersonal Reactivity Index (IRI: Davis 1983). This scale also consists of 7 items on a 5 point Likert-scale ranging from 0 to 4. An example item is “Before criticizing somebody, I try to imagine how I would feel if I were in their place.” The internal consistency was good, ranging from .77 to .80 on the different waves, except for the first two waves, in which Empathic Concern had a relatively low Cronbach’s alpha of .62 for the first wave and .66 for the second wave.

#### Childhood trauma

The Dutch version of the Childhood Trauma Questionnaire-Short Form (CTQ-SF: Thombs et al. 2009) was completed at the 9^th^ wave of data collection to retrospectively assess adolescents’ experienced frequency of maltreatment. This questionnaire is often used as indicator of early childhood stress (e.g., Vinkers et al. 2015) and consists of five subscales: physical abuse, emotional abuse, physical neglect, emotional neglect, and sexual abuse. Although this questionnaire mainly focuses on parental maltreatment, while other forms of childhood traumas are not taken into account, like peer-victimization, accidents or death of close relatives, it is common to refer to this questionnaire as measuring childhood trauma (Scher et al. 2001). All subscales consist of 5 items, except for the 4-item sexual abuse scale. A fifth item of the sexual abused scale “I believe I was molested” was not included in the Dutch CTQ-SF as there is no proper translation for the word ‘molested’ with a sexual connotation (Thombs et al. 2009). The sum of scores on the 24 items was used as a continuous measure for the level of childhood trauma. An example of an item is “During my youth, I had to wear dirty clothes” (physical neglect). With a Cronbach’s alpha of .85, the internal consistency of this scale was adequate.

#### Psychological control

The Dutch adjusted version of the Psychological Control Scale consisted of 8 items on a 5-point Likert scale ranging from not at all applicable to completely applicable (Barber 1996). Adolescents rated the psychological control of their father and mother separately. An example item is “My mother often interrupts me”. The questionnaires were completed until Wave 7 (age 20) as many adolescents moved out of the parental house during the final waves. The internal consistencies were high, ranging across waves from .84-.89 for psychological control of fathers, with only a relatively lower Cronbach’s alpha of .75 for the first wave, and from .83 to .88 for psychological control of mothers. Correlations between waves for the 6-year interval ranged from .37 to .76 for fathers and from .25 to .67 for mothers. Correlations between ratings of mothers and fathers ranged from .56 to .72 within waves. For each adolescent, a mean score over the several waves and fathers and mothers was calculated, as there was not sufficient power to include it as time-varying covariate.

#### MR haplotypes

To identify the different MR haplotype variants, DNA extracted from saliva samples was genotyped and called with the Affymetrix 6.0 array using standard procedure (McCarroll et al. 2008). Sample with sex mismatch, the Affymetrix CQC < 0.40 or a genotyping calling rate < 0.90 were removed. Another exclusion criterium was if the 10 genetic principal components indicated a Caucasian European (CEU) ethnic outlier after projection of the study samples on the 1000 genomes reference sample. In a second step, single nucleotide polymorphisms (SNPs) were filtered using Plink 1.09 (Purcell et al. 2007). The following criteria were used: No or incorrect mapping on Build 37 HG19 of the human genome, inconsistent calls in plate control samples with an error rate > 1%, < 0.95 genotyping rate, minor allele frequencies (MAF) < 0.01, Hardy-Weinberg equilibrium p > 0.000001. After this quality control (QC), during the third step, all SNPs were strand aligned to the 1000 Genomes phase 1 version of June 2014. Subsequently, the genotype data were phased using SHAPEIT V2.970 (Delaneau et al. 2013) and imputed to the 1000 gnomes reference with IMPUTE 2.3.1 following standard protocols (van Leeuwen et al. 2015).

Two commonly investigated functional SNPs in the gene encoding the MR, rs2070951 and rs5522 (DeRijk et al. 2008), were selected to construct haplotypes (de Kloet et al. 2016). Rs5522 was genotyped and rs2070951 was imputed (Li et al. 2010), with R^2^ values and average call rates > 0.99. Chi-square tests were performed to investigate deviations from Hardy-Weinberg equilibrium (HWE), using the HardyWeinberg package in R (Graffelman 2015). Whereas rs2070951 showed HWE (*p* = 0.45), rs5522 was not in HWE (*p* = 0.003). Subsequently, SNPHAP (Clayton 2004) was used to construct haplotypes: GA (47.7%), CA (35.4%), CG (16.6%) and GG (0.3%). This procedure resulted in the final distribution of CA haplotypes: 41.40% adolescents had 0 CA haplotypes, 46.36% had 1 CA haplotype, and 12.24% had 2 CA haplotypes, with more CA-haplotypes indicating increased MR expression and activity (van Leeuwen et al. 2011). This haplotype also influences MR functionality and is often used to investigate the effects of common functional genetic MR variation in clinical samples (e.g., Hardeveld et al. 2015) and population based samples of adults (e.g., Vinkers et al. 2015).

### Analyses

To predict the development of prosocial behavior, empathic concern and perspective taking over the course of adolescence and young adulthood, growth curve analyses were conducted in Mplus 8 (Muthén and Muthén 2017). An equidistant time difference of 1 was used between waves 1 until 6, and an equidistant time difference of 2 was used between waves 6 until 9, corresponding to the annual measurements until age 18 and the biannual measurements after age 18. This resulted in the following growth factors: 0 for age 13, 1 for age 14, 2 for age 15, 3 for age 16, 4 for age 17, 5 for age 18, 7 for age 20, 9 for age 22, and 11 for age 24. The growth curves were estimated in three steps. During the first step, the growth curve was estimated and the model fit of a growth curve with two latent factors (intercept and slope), three latent factors (intercept, slope, and quadratic slope), and four latent factors (intercept, slope, quadratic slope, and cubic slope) were compared using a Satorra-Bentler scaled Chi Square difference test (Satorra and Bentler 2010). To enhance the interpretation of the effect of the predictor on the developmental trajectory, the best fitting growth curves were transformed into piecewise models with different slopes for each part of development (Flora 2008), as the slopes might be differently related to predictors (Diallo and Morin 2015). Knots or transition points between two slopes were determined at the point where the curves bended, but with at least three time points for each slope as this is important for power and convergence (Diallo and Morin 2015). The fit of these models was evaluated based on the following criteria: acceptable fit when CFI > .90, RMSEA and SRMR < .10, and good fit when CFI > .95, RMSEA < .06 and SRMR < .08 (Kline 2005).

In the second step the main effect of sex was added as predictor of these latent growth factors to estimate sex differences in social development. In the third step, besides sex, stress and MR (0, 1, or 2 CA haplotypes, for an additive genetic model) and all 2-way and 3-way interaction terms were included as independent variables to investigate the direct and moderating role of MR. Six different growth curve models were estimated in which all growth factors (intercept and piecewise slopes) were predicted at the same time, with either parental psychological control or childhood trauma as stress variable and separate models for each indicator of social behavior: prosocial behavior, empathic concern, and perspective taking.

## Results

### Descriptives

Table 1 shows the correlations between the study variables. The three indicators of social behavior were moderately correlated to each other, with slightly higher correlations between the two subscales of the IRI, empathic concern and perspective taking, than between these subscales and prosocial behavior. Correlations of the same subscales between two consecutive waves were moderate to high, indicating relative stability over time. Overall, there were small differences in correlations for boys and girls. If correlations differed significantly between boys and girls, correlations for boys were generally slightly higher. MR was unrelated to all indicators of social behavior. Both parental psychological control and childhood trauma showed a negative relation with indicators of social behavior. Parental psychological control and childhood trauma were positively related (*r* = .46, *p* < .001), whereas the number of MR CA haplotypes was not related to the level of parental psychological control (*r* = −.007, *p* = .897), or childhood trauma (*r* = −.066, *p* = .261).

**Table 1:** Correlation between waves, prosocial behavior (PB), empathic concern (EMP), perspective taking (PET) scales, sex, MR, childhood trauma (CT) and parental psychological control (PC)

	EMP same wave		PET same wave		Same scale next wave		Sex	MR	CT	PC
	Boys	Girls	Boys	Girls	Boys	Girls				
PB1	.45*	.35*	.36*	.18*	.50*	.18*	.22*	−.03	−.13*	−.08
PB2	.36*	.28*	.19*	.15t	.38*	.20*	.37*	−.03	−.14*	−.05
PB3	.26*	.34*	.28*	.29*	.37*	.30*	.33*	−.02	−.22*	−.17*
PB4	.35*	.33*	.24*	.18*	.37*	.38*	.31*	.04	−.16*	−.12*
PB5	.41*	.28*	.28*	.22*	.57*	.39*	.24*	−.04	−.14*	−.13*
PB6	.34*	.26*	.38*	.18*	.41*	.33*	.19*	.04	−.07	−.10t
PB7	.34*	.34*	.29*	.38*	.68*	.52*	.22*	.00	−.16*	−.21*
PB8	.25*	.26*	.33*	.26*	.63*	.56*	.09	−.01	−.25*	−.23*
PB9	.41*	.42*	.34*	.22*			.18*	.02	−.23*	−.15*
EMP 1			.39*	.67*	.39*	.45*	.30*	−.03	−.03	.03
EMP2			.42*	.46*	.52*	.63*	.42*	−.05	−.12*	.00
EMP3			.54*	.66*	.58*	.57*	.48*	−.04	−.06	-.03
EMP4			.61*	.55*	.66*	.64*	.41*	−.04	−.15*	−.08
EMP5			.56*	.47*	.69*	.60*	.39*	−.02	−.10t	−.06
EMP6			.49*	.54*	.65*	.70*	.37*	−.01	−.13*	−.04
EMP7			.47*	.48*	.62*	.64*	.38*	.03	−.09	−.03
EMP8			.51*	.46*	.65*	.57*	.38*	−.06	−.09	−.02
EMP9			.50*	.40*			.36*	.04	−.13*	−.03
PET1					.37*	.44	.10t	.00	−.08	−.07
PET2					.60*	.58	.28*	−.05	−.05	.01
PET3					.59*	.66	.37*	−.06	−.07	−.12*
PET4					.57*	.66	.29*	−.03	−.11t	−.08
PET5					.65*	.73	.23*	−.04	−.11t	−.11*
PET6					.58*	.67	.25*	.05	−.11t	−.07
PET7					.72*	**.58**	.14*	.00	−.12*	−.15*
PET8					.75*	**.55**	.17*	.02	−.10t	−.11t
PET9							.15*	.03	−.13*	−.16*

*Note*. Correlations in bold differ significantly between boys and girls

*t < .10, *p* < .05.

Table 2 shows the descriptives of the study variables for boys and girls separately. Girls scored slightly higher on all indicators of social behavior at all time points as compared to boys, with effect sizes ranging from *r* = .09 to *r* = .48. Boys and girls did not differ on the reported level of parental psychological control (*F*(1, 341) = 2.45, *p* = .118, *r* = .08) or childhood trauma (*F*(1, 291) = .06, *p* = .800, *r* = .01). Moreover, the number of MR CA haplotypes was not different for boys and girls (*F*(1, 341) = .57, *p* = .450, *r* = .04).

**Table 2:** Descriptives of prosocial behavior (PB), empathic concern (EMP), perspective taking (PET), MR, childhood trauma (CT) and parental psychological control (PC) for boys and girls

	Wave	Boys		Girls					
		M	Sd	M	Sd	Df	F	*p*	*R*
PB	1	59.45	8.74	63.45	9.28	1, 330	16.24	<.001	.22
	2	59.09	9.65	66.73	9.38	1, 329	52.42	<.001	.37
	3	60.09	10.28	66.69	8.50	1, 327	38.61	<.001	.32
	4	60.74	9.72	66.88	8.89	1, 326	34.90	<.001	.31
	5	62.62	7.70	66.78	9.08	1, 325	20.10	<.001	.24
	6	62.08	9.48	65.73	9.83	1, 325	11.54	.001	.19
	7	64.60	6.81	67.53	5.94	1, 310	15.98	<.001	.22
	8	65.16	6.40	66.35	7.08	1, 293	2.29	.132	.09
	9	64.17	7.46	66.75	6.00	1, 291	10.22	.002	.18
EMP	1	16.28	3.47	18.57	3.70	1, 338	34.42	<.001	.30
	2	15.85	3.88	19.49	3.87	1, 326	71.49	<.001	.42
	3	15.49	4.03	19.86	3.96	1, 324	95.70	<.001	.48
	4	15.12	4.44	18.92	4.06	1, 325	63.96	<.001	.41
	5	15.78	3.83	19.03	3.78	1, 325	58.95	<.001	.39
	6	15.96	3.80	19.05	3.82	1, 324	52.56	<.001	.37
	7	16.75	3.54	19.66	3.54	1, 310	52.42	<.001	.38
	8	16.79	3.54	19.75	3.62	1, 299	50.56	<.001	.38
	9	17.22	3.85	20.23	3.85	1, 293	44.69	<.001	.36
PET	1	13.99	3.64	14.75	3.81	1, 338	3.44	.064	.10
	2	13.68	3.52	15.97	4.42	1, 326	27.26	<.001	.28
	3	13.36	4.29	16.79	4.42	1, 324	50.01	<.001	.37
	4	14.08	4.26	16.67	4.40	1, 325	28.91	<.001	.29
	5	14.53	4.19	16.62	4.52	1, 325	18.70	<.001	.23
	6	15.15	3.96	17.26	4.30	1, 324	21.10	<.001	.25
	7	15.66	4.09	16.84	4.15	1, 310	6.33	.012	.14
	8	16.24	3.86	17.54	3.62	1, 299	8.86	.003	.17
	9	16.80	4.11	18.09	3.85	1, 293	6.46	.012	.15
MR	5	.73	.70	.68	.64	1, 341	.57	.450	.04
CT	9	1.37	.32	1.36	.34	1, 291	.06	.800	.01
PC	Mean	14.20	3.76	14.89	4.31	1, 341	2.45	.118	.08

### Growth Curves

**Fig. 1:**
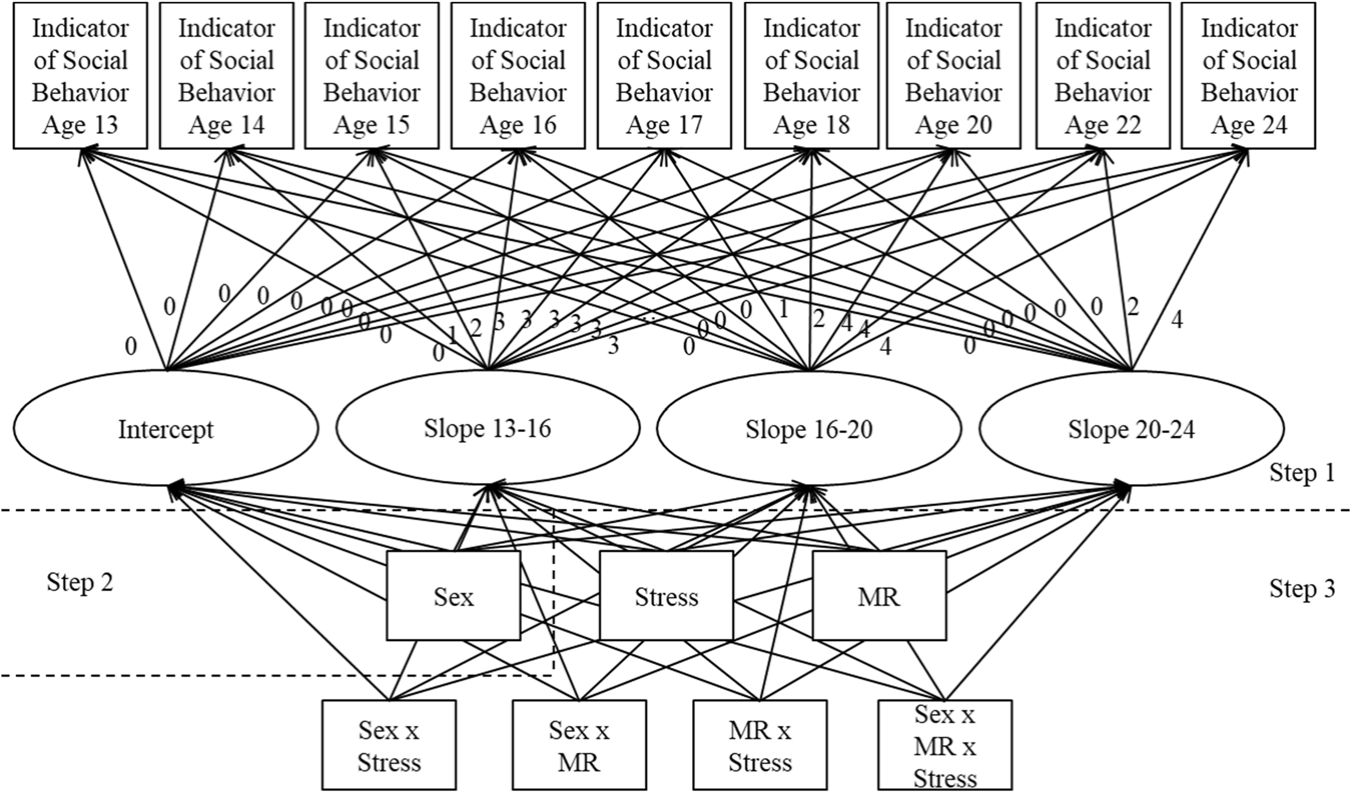
Analytical model of the growth curve estimation. The slope factor loadings were corresponding to the age time scale with a difference of 1 between the ages of 13 until 18, and a difference of 2 between the ages of 18 until 24

The model comparisons of the growth curves with two, three, and four latent factors revealed that model fit of the quadratic model was better than the linear model (∆χ^2^(1.54) = 38.01, *p* < .001 for prosocial behavior; ∆χ^2^(1.18) = 45.85, *p* < .001 for empathic concern; ∆χ^2^(1.20) = 50.67, *p* < .001 for perspective taking), and that the model fit of the cubic model was better than the quadratic model and linear model for all three measures (Prosocial behavior: ∆χ^2^(1.28) = 22.67, *p* < .001 for quadratic, ∆χ^2^(1.40) = 62.72, *p* < .001 for linear; empathic concern: ∆χ^2^(1.18) = 53.38, *p* < .001 for quadratic, ∆χ^2^(1.18) = 99.24, *p* < .001 for linear; perspective taking: ∆χ^2^(1.02) = 81.71, *p* < .001 for quadratic, ∆χ^2^(1.10) = 131.02, *p* < .001 for linear). In the transformation of these cubic models to piecewise models, for all three models the knots were identified at Wave 4 (age 16) and Wave 7 (age 20: see Fig. 1, step 1). The fit of the piecewise models was good (see Table 3, Step 1) and the correlations between latent factors were relatively low (between *r* = −.46 and *r* = .18), indicating that the effects of the predictors on the different latent factors can be reliably estimated.

**Table 3:** Model fit of the growth curve models of the development of prosocial behavior, empathic concern, and perspective taking separately for childhood trauma (CT) and psychological control (PC)

	Prosocial behavior		Empathic concern		Perspective taking	
*Step 1: Growth curve*						
RMSEA	.045		.051		.054	
90% C.I.	.022–.065		.030–.071		.034–.073	
CFI	.951		.981		.976	
SRMR	.052		.059		.070	
*Step 2: Main sex effect*						
RMSEA	.051		.051		.057	
90% C.I.	.032–.069		.032–.069		.039–.075	
CFI	.939		.979		.970	
SRMR	.055		.057		.067	
	**CT**	**PC**	**CT**	**PC**	**CT**	**PC**
*Step 3: All main and interaction terms*						
RMSEA	.032	.033	.026	.038	.054	.048
90% C.I.	.000–.050	.013–.049	.000–.045	.021–.053	.039–.069	.034–.062
CFI	.965	.963	.991	.981	.958	.965
SRMR	.052	.040	.038	.041	.049	.049

### Social Behavior Development and Sex Differences

**Fig. 2:**
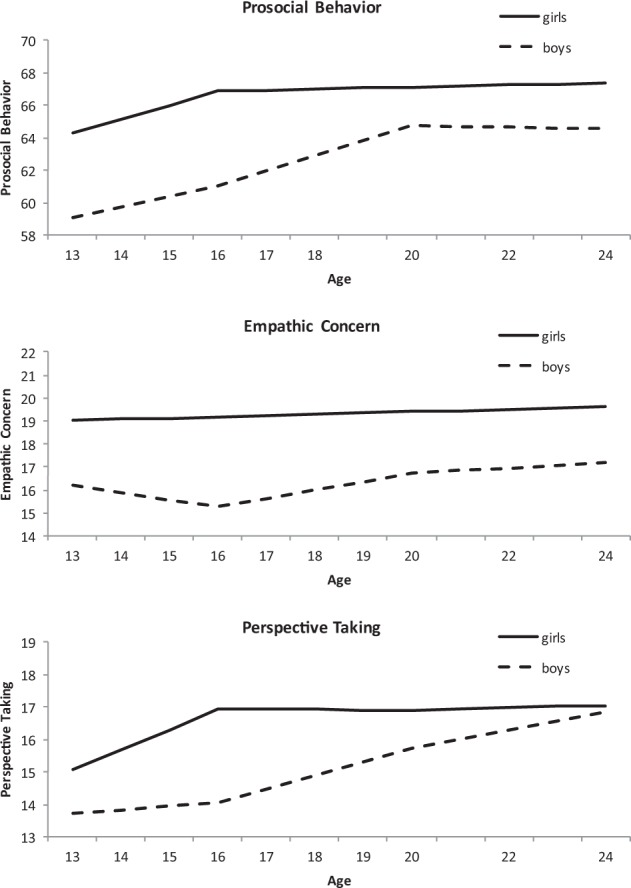
Piecewise model for prosocial behavior (top), empathic concern (middle), and perspective taking (bottom) for boys (dashed line) and girls (straight line)

The growth curves with sex as predictor (see Fig. 1, step 2) generally showed an increase in prosocial behavior, empathic concern and perspective taking for both boys and girls (see Fig. 2, Table 4). Adolescents’ prosocial behavior increased mainly over the course of adolescence, but not over young adulthood, with a higher initial level of prosocial behavior for girls but a stronger increase for boys between age 16 and 20, and no differences between age 13 and 16 or between 20 and 24.

**Table 4:** Intercept and slope factors of the development of prosocial behavior, empathic concern, and perspective taking separately for childhood trauma (CT) and psychological control (PC) and the effect of sex on the growth factors

	General				Sex effect	
	b	*p*	s^2^	*p*	*ß*	*p*
*Prosocial behavior*						
Intercept	61.35	<.001	40.56	< .001	.42	<.001
Slope 13–16 year	7.47	<.001	371.84	.001	.05	.601
Slope 16–20 year	5.49	<.001	117.91	.003	−.40	<.001
Slope 20–24 year	−1.00	.296	85.96	.038	−.06	.541
*Empathic concern*						
Intercept	17.46	<.001	7.59	<.001	.51	< .001
Slope 13–16 year	−1.63	.023	65.49	<.001	.22	.011
Slope 16–20 year	2.34	<.001	33.98	<.001	−.26	.001
Slope 20–24 year	1.29	.008	22.34	.017	.05	.621
*Perspective taking*						
Intercept	14.31	<.001	6.54	<.001	.26	<.001
Slope 13–16 year	3.40	<.001	69.27	.001	.31	<.001
Slope 16–20 year	2.30	<.001	48.40	<.001	−.30	<.001
Slope 20–24 year	2.81	<.001	29.92	.001	.01	.905

Empathic concern slightly decreased in early adolescence, but increased over the course of late adolescence and young adulthood. This developmental pattern was different for boys and girls, with girls showing higher levels of empathic concern at age 13 and a stronger increase until age 16, but a stronger increase for boys between age 16 and 20, and no difference in growth in late adulthood.

Although the cubic model fit also showed the best fit for perspective taking, adolescents significantly increased in perspective taking over the whole course of adolescence and young adulthood. Again, girls showed higher levels of perspective taking at age 13 and a stronger increase in perspective taking between age 13 and 16, whereas boys showed a stronger increase between age 16 and 20, and no difference in growth from age 20 until 24.

In sum, all three models showed an increase in indicators of social behavior over the course of adolescence and young adulthood. Whereas prosocial behavior increased earlier and empathic concern increased later, perspective taking increased gradually over the whole course of adolescence and young adulthood. Girls showed initially higher levels of indicators of social behavior, but boys showed a stronger increase during late adolescence.

### Prosocial Behavior, Stress and MR

**Fig. 3:**
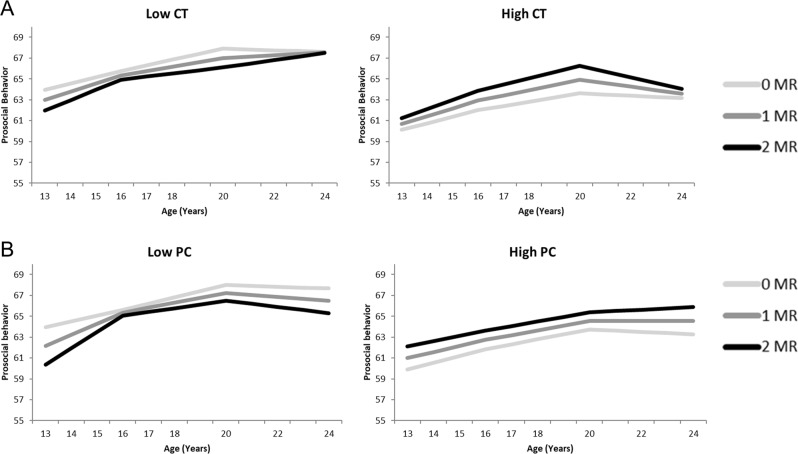
Prosocial behavior growth curve models of both for Childhood Trauma (CT: **A**) as well as Psychological Control (PC: **B**), separately for low (left) and high (right) levels of stressful parenting as plotted for 1 SD above and below average. The darker lines correspond to more MR CA haplotypes, which is indicative of higher MR functioning. Graphs show in general higher levels of prosocial behavior for more MR CA haplotypes (darker lines) in adolescents with high levels of reported stress (High CT and High PC), but lower levels of prosocial behavior for more MR CA haplotypes in adolescents with lower levels of reported stress (Low CT and Low PC)

The model with childhood trauma showed no MR main or interaction effects on prosocial behavior (see Table 5, Fig. 3a). The model with psychological control showed a negative main effect of parental psychological control on the intercept of prosocial behavior, and an interaction effect between parental psychological control and MR on the intercept of prosocial behavior. This indicated that, although adolescents who reported high levels of parental psychological control reported less prosocial behavior, the MR CA haplotype was more protective for the prosocial behavior of adolescents with high parental psychological control as compared to adolescents with lower levels of parental psychological control (see Fig. 3b). The significant interaction effect of MR and stress on the intercept of prosocial behavior in combination with the non-significant (interaction) effects of MR on the slopes of prosocial behavior suggest that the positive role of MR CA haplotypes on prosocial behavior in adolescents who reported high parental psychological control remained over the course of adolescence. In neither the model with childhood trauma, nor the model with psychological control, there were sex-effects, except for a main effect of sex on the intercept of prosocial behavior and on the slope of prosocial behavior between 16 and 20 years, with the latter effect only significant for the model with childhood trauma. These results are in line with the sex differences in the growth model without MR and stress as predictors.

**Table 5:** Standardized regression coefficients of the growth curve models of the development of prosocial behavior, empathic concern and perspective taking separately for childhood trauma (CT) and parental psychological control (PC)

	Prosocial behavior		Empathic concern		Perspective taking	
	CT	PC	CT	PC	CT	PC
*Intercept effects*						
MR	−.06	−.09	−.07	−.11	−.01	−.04
Sex	.38**	.38***	.47***	.42***	.30*	.26*
Stress	−.16	−.43**	−.05	−.15	.14	−.21
MR∗sex	.06	.09	.09	.14	.02	.01
MR*stress	−.04	.38**	−.06	.20t	−.33*	.10
Stress∗sex	−.19	.16	.08	.18	.04	.21
MR∗sex∗stress	.23	−.20	−.03	−.21t	−.05	−.19
*Slope 13–16 effects*						
MR	.07	.11	−.06	−.06	−.05	−.03
Sex	.03	.09	.14	.17	.24t	.31**
Stress	−.08	.10	−.39*	−.36*	−.39*	−.13
MR∗sex	−.02	-.05	.12	.11	−.01	.02
MR∗stress	.00	-.24	.32t	.29t	.48**	.15
Stress∗sex	.13	-.11	−.04	−.04	−.12	−.20
MR∗sex∗stress	−.03	.11	−.01	−.07	.08	.09
*Slope 16–20 effects*						
MR	.00	−.05	.11	.12	.09	.06
Sex	−.35t	−.40**	−.17	−.22t	−.31**	−.32**
Stress	−.10	−.29	.26*	.24	−.15	−.17
MR∗sex	−.01	.00	−.09	−.08	.02	.03
MR∗stress	.28	.12	−.20t	−.29t	.13	.23t
Stress∗sex	.18	.33	−.05	.05	.15	.09
MR∗sex∗stress	−.25	−.10	.08	.12	−.08	−.13
*Slope 20–24 effects*						
MR	−.06	−.02	.18	.17	.03	.08
Sex	−.15	−.10	.27t	.28t	.06	.08
Stress	−.14	.18	−.15	.07	.01	.03
MR∗sex	.10	.04	−.36t	−.34t	−.05	−.11
MR∗stress	−.17	.07	.05	.20	−.01	−.28t
Stress∗sex	.17	−.28	.17	−.24	−.02	−.08
MR∗sex∗stress	−.07	.08	−.14	−.12	.01	.33t

*t* < .10; *<.05; **<.01; ***<.001.

### Empathic Concern, Stress and MR

**Fig. 4:**
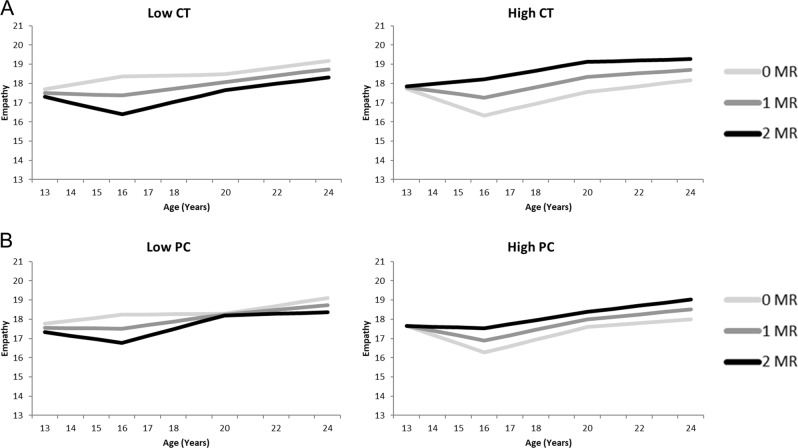
Empathic concern growth curve models of both for Childhood Trauma (CT: **A**) as well as Psychological Control (PC: **B**), separately for low (left) and high (right) levels of stressful parenting as plotted for 1 SD above and below average. The darker lines correspond to more MR CA haplotypes, which is indicative of higher MR functioning. Graphs show in general higher levels of empathic concern for more MR CA haplotypes (darker lines) in adolescents with high levels of reported stress (High CT and High PC), but lower levels of prosocial behavior for more MR CA haplotypes in adolescents with lower levels of reported stress (Low CT and Low PC)

Results of the model with childhood trauma (see Table 5, Fig. 4a) showed a slower increase in empathic concern between age 13 and 16 for adolescent who reported higher levels of childhood trauma, but a stronger increase in empathic concern between age 16 and 20. The empathic concern model with parental psychological control showed a comparable main effect for psychological control as the model for childhood trauma (see Fig. 4b), with a slower increase between age 13 and 16 for adolescents who reported higher levels of psychological control. Both models showed multiple marginally significant interaction effects between MR and stress pointing towards a protective role of MR CA haplotype for the development of empathic concern over the course of adolescence for adolescents with high levels of childhood trauma or psychological control. In addition to a main effect of sex on the intercept of empathic concern indicating higher levels of empathic concern for girls as compared to boys, there was an almost significant interaction effect between MR and sex on the slope between age 20 and 24 (*p* = .050 and *p* = .051 for childhood trauma and psychological control respectively). This suggests that during this period, empathic concern increased more in boys with more MR CA haplotypes than in boys with fewer MR CA haplotypes, while the opposite pattern was seen in girls.

### Perspective Taking, Stress and MR

**Fig. 5:**
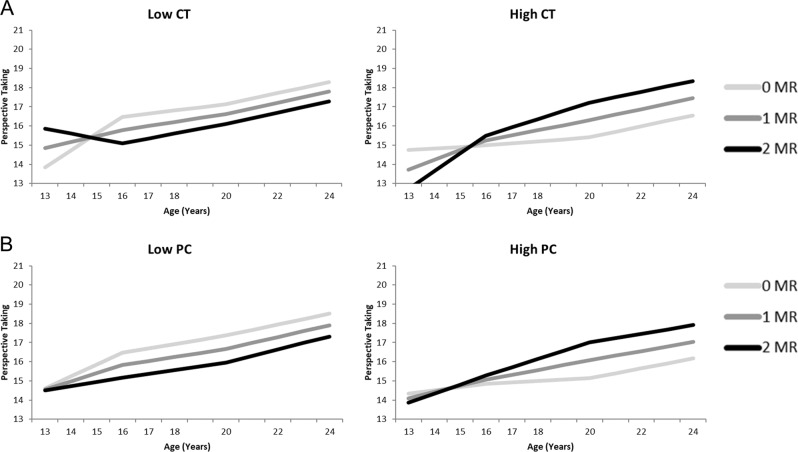
Perspective Taking growth curve models of both for Childhood Trauma (CT: **A**) as well as Psychological Control (PC: **B**), separately for low (left) and high (right) levels of stressful parenting as plotted for 1 SD above and below average. The darker lines correspond to more MR CA haplotypes, which is indicative of higher MR functioning. Graphs show in general higher levels of perspective taking for more MR CA haplotypes (darker lines) in adolescents with high levels of reported stress (High CT and High PC), but lower levels of prosocial behavior for more MR CA haplotypes in adolescents with lower levels of reported stress (Low CT and Low PC)

Results of the model with childhood trauma as stress factor showed a slower increase in perspective taking between age 13 and 16 for adolescents with higher levels of childhood trauma (see Table 5, Fig. 5a). Also, the interaction between MR and childhood trauma on the intercept and slope between age 13 and 16 of perspective taking was significant. Adolescents with high levels of childhood trauma and more MR CA haplotypes initially scored relatively low on perspective taking at age 13, but showed a stronger increase between age 13 and 16 resulting in higher levels of perspective taking at age 16 as compared to adolescents with fewer MR CA haplotypes. Adolescents who reported low levels of childhood trauma and more MR CA haplotypes showed the opposite pattern: they initially had higher levels of perspective taking but showed less increase between age 13 and 16 as compared to adolescents with fewer MR CA haplotypes. As there were no significant main or interaction effects of MR on the slopes of perspective taking after age 16, the stimulating role of MR CA haplotype for perspective taking during early adolescence in adolescents who reported high levels of childhood trauma seemed to remain over the course of late adolescence and young adulthood. Results of the perspective taking model with parental psychological control as stress factor was comparable (see Fig. 5b), but with only a marginally significant interaction effects of psychological control x MR effect on the slope of perspective taking between age 16 and 20 (*p* = .070) in combination with a marginally significant interaction effect on the slope between age 20 and 24 for psychological control x MR (*p* = .070) and for sex x psychological control x MR (*p* = .064). These findings suggest that for adolescent who experienced high levels of psychological control, mainly during late adolescence and early adulthood MR CA had positive effects on their perspective taking development. Besides, in both the model with childhood trauma and psychological control there were significant main effects of sex on the intercept and on the slopes of perspective taking between age 13 and 16 (although only marginally significant for childhood trauma) and between age 16 and 20. These results are in line with the sex differences in the growth model without MR and stress as predictors.

## Discussion

It recently has become clear that the mineralocorticoid receptor (MR) plays a role in stress responsiveness by balancing glucocorticoid levels in the brain (Joëls, Karst, DeRijk, and de Kloet 2008), resulting in changes in social behavior (van Leeuwen et al. 2011). However, a couple of limitations in this body of literature constrain its implications. Studies so far have addressed the role of MR in socio-cognitive behaviors in adult samples, clinical samples and have used experimental designs to manipulate the level of MR expression (e.g., Wingenfeld et al. 2014). However, effects of MR on social behavior might emerge in an age period when this development is strong, like adolescence, and therefore longitudinal studies are needed to examine when this association emerges. Also, for preventive purposes, consequences of natural variation in the MR gene in a community sample are important as well. Moreover, both in the MR field (ter Heegde et al. 2015) and in the social development literature (van der Graaff et al. 2018) sex-differences are pronounced, but both theoretically and empirically, it is unclear how these sex-specific factors can interact. Therefore, this longitudinal community study examined how the development of prosocial behavior, empathic concern, and perspective taking during adolescence and young adulthood was affected by stressful parenting and common genetic variation in the MR gene in boys and girls. In individuals who experienced higher levels of parental psychological control or childhood trauma, the number of CA haplotypes was positively associated with social development. This protective effect of the MR CA haplotype emerged over the course of adolescence and young adulthood for empathic concern and perspective taking. Although social development was different for boys and girls, with a later increase in prosocial behavior, empathic concern, and perspective taking for boys, there was no difference in the role of the MR CA haplotype between boys and girls in this developmental pattern.

Adolescents who likely experienced high levels of stressful parenting, as indicated by parental psychological control or childhood trauma, showed lower levels of, or a slower growth in, prosocial behavior, empathic concern, and perspective taking skills. Childhood trauma had more significant relations with the social development than parental psychological control, possibly as childhood trauma have been more extreme stressors as compared to parental psychological control. It also might be explained by the stressful event load that accumulates during lifetime (Kemner et al. 2015). Childhood traumas might have happened at a younger age and therefore had more cumulative effect, as the Childhood Trauma Questionnaire is a retrospective measure about adolescents’ whole youth until the age of 18, while parental psychological control was measured parallel to social development during adolescence from age 13. We were not able to establish time order between stressful parenting and changes in social behavior and can only conclude that reported trauma or parental psychological control is related to change in social behavior. Still, both forms of stressful parenting might have the same kind of consequences. When parents are psychologically controlling, they display disappointment and children feel pressured and guilty that they did not comply with the parent’s requests or feels anxious about losing the parent’s approval (Soenens and Vansteenkiste 2010). Also, childhood abuse is characterized by guilt and self-blame of the child (Valle and Silovsky 2002). These processes may result in a stronger focus of adolescents on the consequences of their behavior for themselves, by complying to their parents, as compared the needs of others (Hoffman 1983). Future studies might provide more insight in this process by asking participants to report the level stress associated with childhood trauma or parental psychological control, and asking the exact timing and duration of these stressful experiences.

There were no main effects of MR CA haplotype for social development, but there were interaction effects between MR and stressful parenting. Overall, the MR CA haplotype had positive effects on social development of adolescents who reported high levels of parental psychological control or childhood trauma, and even seemed to have negative effects for adolescents with low levels of stressful parenting, although these effects did not reach significance in all models. This suggests that adolescents do not differ in their general susceptibility to stress based on their MR haplotype, but that the MR CA haplotype has stronger positive effects for higher levels of stress. This pattern is in accordance with the study of Vinkers et al. (2015) that found stronger MR CA haplotype effects for higher levels of childhood trauma, resulting in less depression symptoms. The opposite pattern for adolescents with low levels of stressful parenting in combination with MR CA haplotype, resulting in relatively less positive social development, fits within the differential susceptibility theory (Pluess 2015). This theory supposes that sensitive individuals are vulnerable for developing certain outcomes, but whether the environment is adverse or supportive results in vulnerability or enhancement. In light of this theory, fewer MR CA haplotypes can be seen as an indicator of sensitivity, resulting in less positive social development for adolescents with high levels of stressful parenting and better social development for adolescents with supportive parents. Having more MR CA haplotypes is an indicator of resiliency, making adolescents with more MR CA haplotypes equally social in both stress and non-stress environments. The good social development for adolescents with high stressful parenting and more MR CA haplotypes even seemed to outreach the social development of adolescents with low levels of stressful parenting and more MR CA haplotypes, suggesting that people with more MR CA haplotypes thrive better in a stressful environment. Important to note is that the role of the MR CA haplotype in interaction with their level of stressful parenting was less pronounced for empathic concern, and although patterns for both parental psychological control and childhood trauma were comparable, the results were not always significant for both forms of abusive parenting. Until now, too little is known about the underlying biological mechanisms of MR in positive and negative environments (see ter Horst et al. 2014). Further research is needed into the interaction between environment and MR expression to better understand the consequences of the interaction between high and low levels of stressful parenting and genetic variation in the MR gene for adolescents’ prosocial behavior, empathic concern and perspective taking.

The positive role of the MR CA haplotype (in interaction with stress) for indicators of social behavior corresponds with earlier studies in which MR was pharmacologically stimulated and resulted in increased empathic concern (Wingenfeld et al. 2014) and emotion processing (Schultebraucks et al. 2016). But earlier studies found no relation between MR CA haplotypes and perspective taking (Wingenfeld et al. 2014), or even diminished perspective taking (Wingenfeld et al. 2016). In these studies, perspective taking abilities were measured directly after the pharmacological manipulation. Until date it is unclear how direct consequences of MR genetic variation differ from long term consequences, and acute stress from chronic stress (Vogel et al. 2016). Possibly, the long-term behavioral consequences of stress and MR genetic variation are larger as compared to the short-term behavioral consequences, as stress affects the development of several brain areas, like the prefrontal cortex (McEwen et al. 2016). The prefrontal cortex is relevant in social behavior as it provides top-down control (Arnsten et al. 2015). Thereby, for adolescents who experienced stress, social behavior could be affected via a different prefrontal cortex development. To better understand the role of MR in the relation between stress and social development, more research is needed into the role of MR in prefrontal cortex functioning and into the development of the prefrontal cortex under stress.

The MR CA haplotype mainly played a role (in interaction with stress) over the course of adolescence for the development of empathic concern and perspective taking, while for prosocial behavior the effects of the MR CA haplotype were already evident at the beginning of adolescence. The period at which MR CA haplotype affected the indicators of social behaviors most, corresponds to those points during adolescence at which these behaviors showed the main development. During these so called ‘developmental switch points’ (Ellis et al. 2013), there is increased susceptibility to genes and environmental influences (Del Giudice et al. 2011).

The hypothesis that mainly women with more MR CA haplotypes were relatively more social was not confirmed. This is in contrast to rodent studies that found female-specific MR effects in social discrimination (ter Horst et al. 2014) and emotional behaviors (ter Horst et al. 2012) and a human female-specific protective effect of MR CA haplotype for the development of depression (Klok et al. 2011) even as moderator of childhood stress (Vinkers et al. 2015). These effects are generally explained by the female hormones, estrogen and progesterone that affect MR functioning, although the exact underlying mechanism is unclear (Carey et al. 1995). Evidence for a sex-specific MR effect did not come from adolescent studies. Adolescent girls may respond differently to stress as compared to woman, as during adolescence, the increasing levels of female hormones levels influence neurotransmitter systems which affect the maturing HPA axis (Naninck et al. 2011). More research is needed about the interaction between MR and female hormones during adolescence versus adulthood to better understand the sex-specific role of MR for different behaviors.

Looking at the development of the different indicators of social behavior, whereas perspective taking showed a relatively gradual increase over the whole course of adolescence and young adulthood, prosocial behavior mainly increased during early adolescence, and empathic concern increased during late adolescence and young adulthood. These findings are consistent with earlier studies on social development. Earlier longitudinal studies of prosocial behavior have also found an increase in early adolescence (Fabes et al. 1999), and no development in late adolescence (Flynn et al. 2015) or young adulthood (Eisenberg et al. 2005). Studies on empathic concern (Brouns et al. 2013) and perspective taking mainly showed an increase during late adolescence (Eisenberg et al. 2005). These differences in developmental pattern for prosocial behavior versus empathic concern and perspective taking are likely due to prefrontal cortex functioning. During adolescence, the prefrontal cortex rapidly develops (Blakemore 2008), which is supportive for taking one’s own and others’ perspective, and regulation of behavior, thought and emotion (Arnsten et al. 2015). Perspective taking and empathic concern rely more on these skills as compared to prosocial behavior (Shamay-Tsoory et al. 2009), which might explain why perspective taking and empathic concern develop until later as compared to prosocial behavior.

In line with other studies, sex differences were found in social development. Boys showed lower levels of social skills as compared to girls, and a later increase in all social skills as compared to girls. Other studies have also found higher levels of social skills in girls (Miklikowska et al. 2011) and an earlier development of empathic concern for girls during adolescence (Carlo et al. 2015). But developmental differences in prosocial behavior between boys and girls have rarely been studied and there are also studies showing a more rapid decline for the prosocial behavior of boys (Carlo et al. 2007). The current pattern of findings, with an earlier growth for girls as compared to boys, has already been reported by van der Graaff et al. (2018), conclusions whom were derived from the first 6 waves of the current data set. A process that may account for these sex differences is gender role expectations. Due to gender role identification, which tends to be strongest during early adolescence (Eagly 2009), girls show higher levels of social behaviors because they are stimulated to show nurturance and care.

This study had some limitations. First, the sole focus on hypothesis-driven MR as a CA haplotype, instead of MR expression, MR functioning, or even the broader stress system, gives only a limited view of how the stress system performs in relation to social behavior. More research that examines the whole stress-axis is needed to better understand the biological mechanism behind the role of MR for social behavior. Second, we included genetic influences and parental influences on social development, although parenting might be confounded with heritability of the MR genes (i.e. passive gene-environment correlation). This might suggest that parents who have better stress regulation show relatively less stressful parenting towards their child, and at the same time have children with a MR haplotype that makes them better able to regulate the stressful parenting. However, in our study we did not find a relation between MR haplotype and stressful parenting, which makes it unlikely that heritability of MR-genes would have confounded our findings. Moreover, given the hypothesis driven set-up of this study, there was no independent sample taken into account to replicate the findings, while this is currently standard in gene-environment studies. It is of note that one of the SNPs, rs5522, was not in HWE and that we cannot exclude possible genotyping errors. Importantly, however, a previous study showed that approximately 10% of genotype-phenotype associations deviated from HWE (Trikalinos et al. 2006). Another limitation is the use of questionnaires to let adolescents report on indicators of social behavior, with possible differences between their actual social behaviors and the reports of their social behaviors. It would have been better if experimental tasks were added to measure their actual prosocial behavior and cognitive and empathic concern. Moreover, the questionnaire on childhood trauma was retrospective, which may limit the validity of the reports as it may have been subject to recall bias. Still the questionnaire resulted in reliable reports in multiple studies, amongst others in a community based sample from 18-65 years, with even reliable reports in the age group of 45-65 years (Scher et al. 2001). Also, a composite score for psychological control was calculated over fathers, mothers and seven waves, with possibly interesting information being lost. Finally, a limitation is the use of six different growth models, resulting in multiple testing and possibly false positives. On the other hand, the effect sizes are quite large, and by estimating separate models the results can be better related to previous studies on these different aspects of social functioning. Further research into the consequences of stressful parenting on social behavior, and the biological underlying mechanism, with larger sample sizes, is needed to better understand the exact role of MR for social development in highly and lowly stressed adolescents.

## Conclusion

Adolescence is characterized by rapid social development (Tousignant et al. 2017), but is also the period in which long-lasting effects of stress become evident (Lupien et al. 2009), possibly affecting this social development negatively (Sandi and Haller 2015). One of the biological factors that could play a role in this process is the mineralocorticoid receptor (MR). Functional gene variation, more specifically the MR CA haplotype, is associated with higher levels of MR expression, which supports stress regulation and could therefore be advantageous for social behavior (van Leeuwen et al. 2011). Experimental studies have shown that pharmacological stimulation of MR resulted in enhanced socio-cognitive behaviors (Wingenfeld et al. 2014). Unclear is how common functional variation in the MR gene affects social development during adolescence in a community sample. The current study showed that MR CA haplotype moderates the effects of stressful parenting and sex on social development (i.e., prosocial behavior, empathic concern and perspective taking development) from adolescence to young adulthood. Common functional variation in the MR gene resulted in better social development for adolescent that experienced high levels of stressful parenting. These findings are in line with earlier adult studies and studies in clinical samples, making the earlier MR findings more generalizable to the general population. Moreover, the current study showed that the MR effects were evident from the beginning of adolescence, suggesting that already in childhood MR is probably important for the consequences of stress on social development. MR gene variation seems to contribute partially to adolescents’ resiliency to the consequences of stressful parenting, and explains why some adolescents develop socially well despite a stressful childhood, while others show profound social problems and need specialized care.
